# Hallermann-Streiff syndrome with uncommon ocular features, ultrasound biomicroscopy and optical coherence tomography findings: A case report

**DOI:** 10.1097/MD.0000000000018272

**Published:** 2019-12-10

**Authors:** Wei Shen, Min Dai, Yunshan Su, Qing Zhang, Hongsong Li

**Affiliations:** a Department of Ophthalmology, Fourth Affiliated Hospital of Kunming Medical University (the Second People's Hospital of Yunnan Province), Yunnan Eye Institute, Key Laboratory of Yunnan Province for the Prevention and Treatment of ophthalmology, Provincial Innovation Team for Cataract and Ocular Fundus Disease, The Second People's Hospital of Yunnan Province; Expert Workstation of Yao Ke; b Department of Radiology, the Second People's Hospital of Yunnan Province; c School of Information Science and Engineering, Yunnan University, Kunming, China.

**Keywords:** anatomy, embryology and development, Hallermann-Streiff syndrome, image, optical coherence tomography, ultrasound biomicroscopy

## Abstract

**Rationale:**

Hallermann-Streiff syndrome (HSS) is a rare congenital disorder characterized by craniofacial malformations, sparse hair, degenerative skin changes, eye abnormalities, dental defects, and proportionate short stature.

**Patient concerns:**

A 24-year-old Chinese male patient presented to the ophthalmologist because of his sore eye and blurred vision.

**Diagnoses:**

The final diagnosis of presented case is HSS having the main features of the syndrome, however, associated with uncommon ocular features, ultrasound biomicroscopy (UBM) and optical coherence tomography (OCT)changes, including aphakia, glaucoma, long eye axes, cilliary abnormalities, and chorioretinal atrophy.

**Interventions:**

Antiglaucomatous medical therapy failed to reduce the pressure in the right eye and a cyclocryotherapy was carried out. The antiglaucoma eye drops was continued in the left eye.

**Outcomes:**

The intraocular pressure has been reduced to the normal range, but the vision has not improved.

**Lessons:**

In the diagnosis of HSS, we should not ignore the extraordinary information especially uncommon ophthalmic features, UBM and OCT changes. We highlight the necessity of a multidisciplinary approach for accurate diagnosis and appropriate management.

## 1 Introduction

Hallermann-Streiff syndrome (HSS) is a rare congenital disorder of unknown etiology characterized by craniofacial malformations, sparse hair, degenerative skin changes, eye abnormalities, dental defects, and proportionate short stature.^[1–4]^

HSS was first described by Aubry in 1893, and described completely by Hallermann in 1948 and then by Streiff in 1950. The diagnostic criteria reviewed by François in 1958^[1]^ are useful in the diagnosis of HSS, and it is easy to differentiate HSS from other diseases by noting the presence of congenital cataracts associated with mandibular hypoplasia producing a bird-like face at birth. Until 1981, around 150 cases have been reported.^[4]^

Most HSS cases occur sporadically, while a few familial cases have been reported in the literature. The inheritance pattern of HSS is still unknown and the etiology still obscure. Schanzlin et al^[5]^ and Pugliese et al^[6]^ reported a chromosomal defect associated with this syndrome. Gerinec et al^[7]^ reported this syndrome in two generations. Autosomal recessive and autosomal dominant inheritance patterns with de novo mutations have been implied in HSS development. There is no sex predication. The potential causes of HSS include an asymmetric second bronchial arch defect that arise during the fifth or sixth gestational week, maternal infections, paternal age and toxin exposure.^[2]^

An interesting case of Hallermann-Streiff syndrome in a 24-year-old Chinese male patient is reported here, with the emphasis on the ocular findings and supplementary examinations.

## 2 Case report

**Figure 1:**
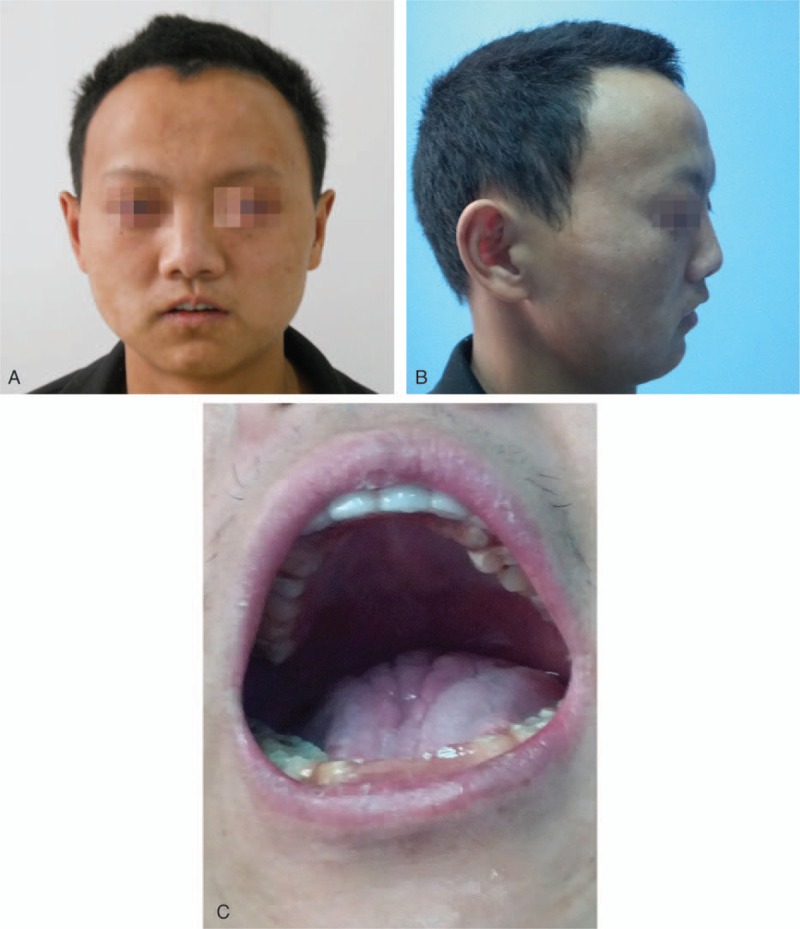
Showing craniofacial malformations, sparse hair, degenerative skin changes, and dental defects of the patient. (A) Full face. (B) Profile. (C) Showing malformed teeth and dental prosthesis in maxillomandibular anterior teeth.

A 24-year-old Chinese male patient presented to the ophthalmologist because of his sore eye and blurred vision. He weighed 45 kg and his height was 150 cm. The head was brachycephalic with prominent frontal and parietal eminences. There was sparse, black hair limited to the posterior two-thirds of the skull. The skin was dry and desquamative. There was marked bird-like face, including micrognathia and considerable symmetrical limitation of movement in the temporo-mandibular joints (Fig. 1). The mouth was small with thin lips and a short upper lip. The palate was highly arched and the tongue was of normal size. He has been fitted dental prosthesis in maxillomandibular anterior teeth by dentists because of partial anodontia (Fig. 1). Note the anterior open bite as well as posterior yellow teeth were. Facial, pubic and axillary hairs are sparse. His motor and mental were retarded mildly.

**Figure 2:**
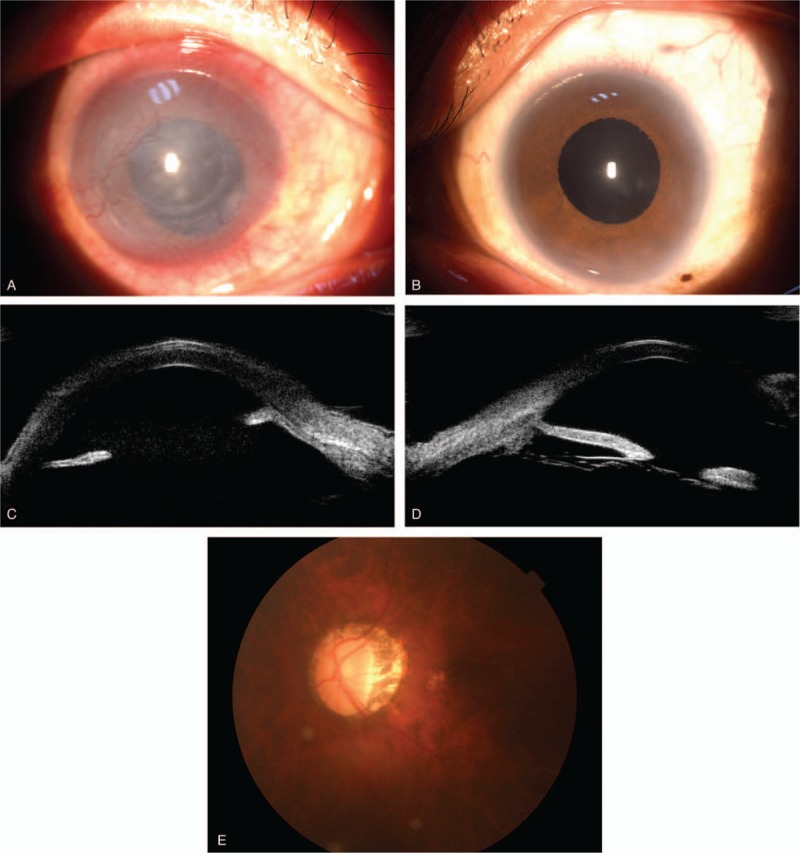
(A) The view of the anterior segment of the right eye showing corneal haze and pannus, and dilated pupils. (B) The view of the anterior segment of the left eye showing absence of lens. Ultrasound biomicroscopic images showing absence of lens and cilliary abnormalities. (C) The right eye. (D) The left eye. (E) The fundus photograph showing a pale and cupped optic disc in the left eye.

The following features were noted in the eyes (Fig. 2): There was no light perception on the right eye and 0.15 in the left eye. Best corrected visual acuity was not improving in the left eye. The intraocular pressure (IOP) was 43 mmHg in the right eye and 35 mmHg in the left eye. The eyelids were lax. Eyebrows and eyelashes were sparse. He had a constant searching, pendular nystagmus in the horizontal gaze but extrocular muscle motion was full. Bilateral microcornea was present. The right cornea was edematous, haze, and pannus. The patient had never undergone cataract surgery, but he was aphakic without any capsular remants bilaterally. The state of the right cornea precluded any further assessment of the eye. The left anterior chamber was deep centrally. Gonioscopic examination revealed that the drainage angle was open. The left fundus was noticed with a pale and cupped optic disc (0.8) and peripapillary choroid atrophy and chorioretinal atrophy. A-scan ocular ultrasonography showed increased axial length of 25.08 mm (right) and 24.68 mm (left) with normal anterior chamber depth (3.39 mm (right) and 3.14 mm (left)), while the horizontal corneal diameter was 9.5 mm (right) and 9.5 mm (left). Keratometry was steep; the right eye was K1 52.57 at 92° and K2 62.62 at 2°; and left eye was K1 53.32 at 114° and K2 55.97 at 24°. Ultrasound biomicroscopy (UBM) showed absence of lens, and did not show normal cilliary process and suspensory ligament in both eyes. The ciliary processes were far forward and even not being viewed (Fig. 2). Optical coherence tomography (OCT) showed thinned peripapillary retinal nerve fiber layer.

**Figure 3:**
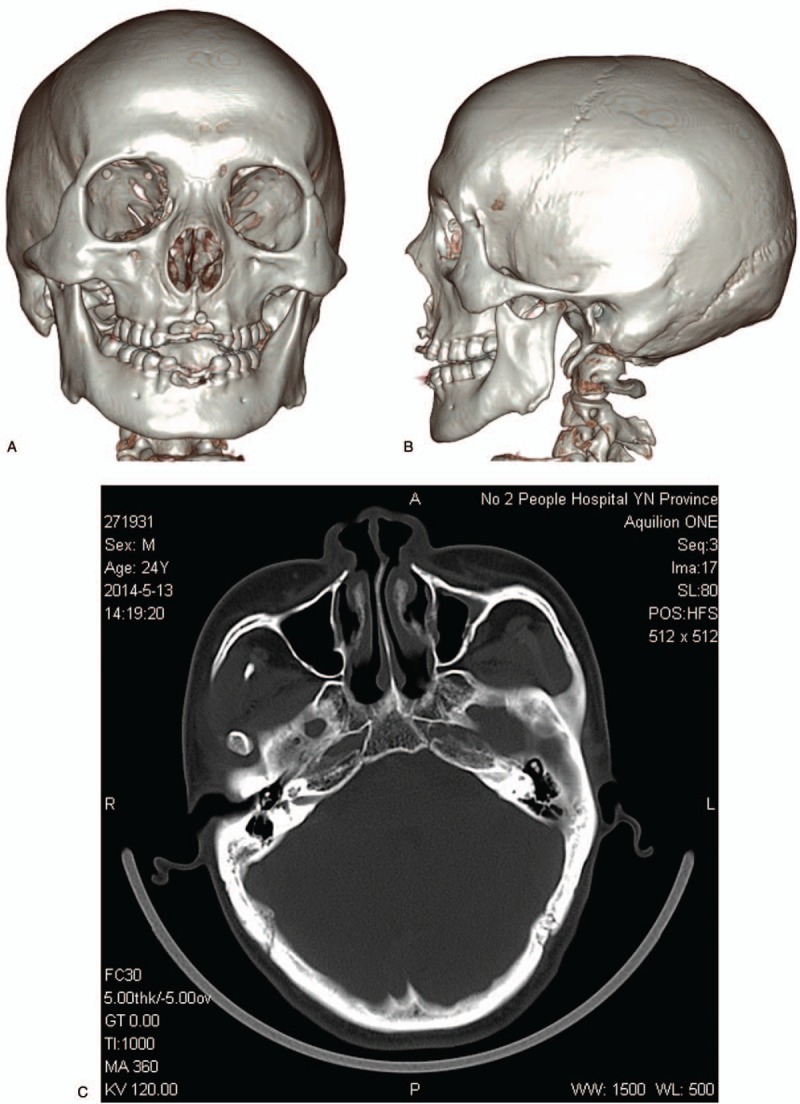
3D reconstructed images of the face showing mandibular deficiency, anodontia and hypoplastic midface with a retrognathic pattern. (A) Full face; (B) Profile. (C) Computer tomography showing a prominent deviation of the nasal septum.

Multi slice computer tomography (MSCT) reconstruction revealed a disproportion between the large cranial vault and the small facial skeleton. The facial skeleton was characterized by a marked posterosuperior positioning of the maxillomandibular complex. Most striking was mandibular deficiency. The ascending ramus and body of the mandible were severely underdeveloped. The prominent deviation of nasal septum was found. Odontogenic anomalies consisting of anodontia in incisor teeth and hypoplastic teeth were also found (Fig. 3).

**Figure 4:**
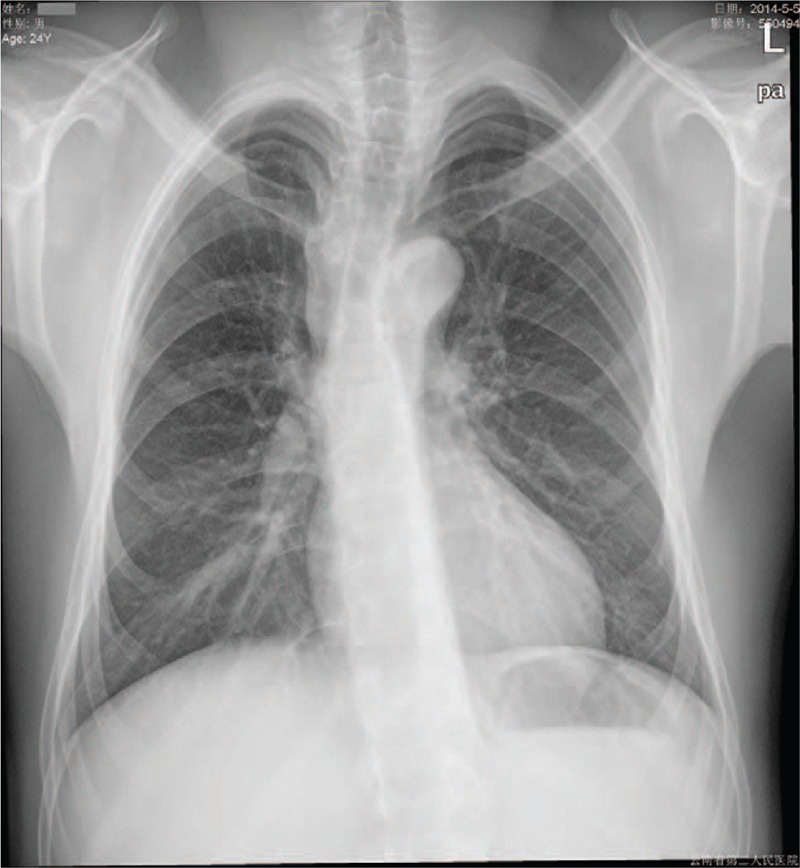
Chest X-ray film showing scoliosis, mild platspondyly, and osteoporosis of thoracic vertebral body.

Chest X-ray film showed scoliosis, mild platspondyly, and osteoporosis of thoracic vertebral body (Fig. 4).

General laboratory studies of blood and urine were normal. No cardiovascular abnormality was present with normal electrocardiography and echocardiography. His chromosome analyses were normal.

Both non-consanguineous, unaffected parents had the age of 24 at the birth of the propositus. The prenatal course, labor and delivery were apparently normal. There were 3 normal sibs, an older and a younger sister, and a younger brother; the mother denied having had miscarriages. The patient had given birth to 2 normal children.

The patient had visited a local hospital and the diagnosis and treatment were unknown. Local doctors recommended transferring to a better hospital. The final diagnosis of presented case is HSS having the main features of the syndrome, however associated with uncommon ocular features, ultrasound biomicroscopy and optical coherence tomography changes, including aphakia, glaucoma, long eye axes, cilliary abnormalities, and chorioretinal atrophy.

Several interdisciplinary doctors were invited to consult about the case. They thought that no need to deal with the situation but for eyes.

The patient was provided with antiglaucoma eye drugs. Antiglaucoma eye drugs (timolol, bromonidine, brinzolamide, and methazolamide) failed to reduce the pressure in the right eye and a cyclocryotherapy was carried out. Continued use of the 3 antiglaucoma eye drops (timolol, bromonidine, and brinzolamide) was prescribed in the left eye. The IOP in the right and left eyes were 11 mmHg and 18 mmHg, respectively. The vision has not improved.

A follow-up examination performed 3 month after surgery on the right eye indicated that visual acuity of the patient remained unchanged and the IOP in the right and left eyes were 10 mmHg and 16 mmHg, respectively. No adverse and unanticipated events were observed. He could not come to our clinic regularly because he resided in a village far away from Kunming, Yunnan.

## 3 Discussion

The case report is of heuristic value for several reasons. HSS is a rare congenital syndrome of unknown etiology. We report a case with HSS having the main features of the syndrome, however, associated with inconstant ocular features, including absence of lens, glaucoma, long eye axes, cilliary abnormalities, and atrophic chorioretinal changes. However, a few new or rare founding were noted that will be discussed below.

The craniofacial findings reported in this case were common features observed in most HSS cases, which include dyscephaly, hypoitrichosis, cutaneous atrophy, ocular abnormalities, and dental abnormalities. Additionally, musculoskeletal abnormalities we observed in this case include proportion short stature, scoliosis, osteoporosis and deformation of thoracic vertebral body, and hyper extensible joint.

Ocular abnormalities are major problems, with the most common features of the eyes being bilateral microphthalmia, congenital cataract, ocular nystagmus, and strabismus. In addition to these principal anomalies, many others have occurred in individual cases; these have been described.^[1,8–14]^

This case showed bilateral absence of lens. In several cases reported this observation with or without capsular remnants occurred. Pasyanthi et al reported 3 cases had a membranous cataract.^[13]^ Bilateral aphakia might have developed as a result of spontaneous absorption of the congenital cataract in the patient. In several of the cases reported, spontaneous resorption of the cataracts occurred.^[11]^ Rohrbach et al reported a case whose spontaneous lens absorption occurred slowly and completely between the beginning of the 2nd and the end of the 3rd year of life without considerable inflammatory responses.^[15]^ They would recommend awaiting the spontaneous lens absorption in the HSS especially when the cataract is combined with considerable microphthalmia. Srivastava et al also reported a case that showed a complete absence of the lens in both eyes. They speculated that the lens was absent from birth.^[16]^

Glaucoma is uncommon for patients with HSS reported in the literature.^[9–12]^ The abnormalities which are likely to cause glaucoma in HSS have 2 possible origins. They may be primary hypogenesis or secondary change resulting from intraocular inflammation. Ludwig and Korting described malformation of the anterior chamber angle of a patient with HSS and glaucoma.^[9]^ Some reports suggested that the intraocular inflammation may have been due to hypersensitivity to the cataractous lens substance derived from spontaneous lens absorption or surgical discussion. It may take a long time to absorb the cataractous lens substance spontaneously unless cataract surgery is performed. The eyes with spontaneous lens absorption may tend to become glaucomatous. However, cataract extraction does not avoid the hazard of glaucoma. A few eyes which underwent cataract surgery also became glaucoma.^[12]^

Most of the reported cases had microphthalmia. We report the case that had longer eye axial length, which is rare in HSS, but did have micocornea and steep keratomethy. Donders PC reported a case whose both eyes were not microphthalmic.^[17]^

UBM can be used to examine the cilliary of eyes that are not observable with conventional optical instruments. Cilliary abnormalities observed with UBM were unreported in HSS. In our patient, the ciliary processes were far forward and even not being viewed. This suggests that there may be a retardation in development, or a progressive degeneration of a ciliary processes caused by loss of lens, that has not been reported in HSS. Unfortunately, the patient was not diagnosed in earlier years. We cannot understand this change come from. Not sure is this is specific for HSS.

Changes on OCT have not been documented in HSS with glaucoma previously. Thinned peripapillary retinal nerve fiber layer may be associated with glaucoma or long eye axes, so this need not to be specific for the Hallermann-Streiff syndrome. In several reported cases in which the fundi have been inspected, degenerative disease of the retina has been noted such as chorioretinal atrophy or peripapillary choroid atrophy.

Productive capacity has not been studied in HSS patients. Our case had given birth to two normal children. A patient of Hendrix SL, a woman with HSS, was artificially inseminated, and a term gestation ensued.^[18]^

In conclusion, our case report provides additional information especially uncommon ophthalmic features, UBM and OCT changes, to the previous literature on HSS. We emphasize the presence of uncommon ophthalmic features of HSS, including aphakia, glaucoma, long eye axes, cilliary abnormalities, and chorioretinal atrophy. We highlight the necessity of a multidisciplinary approach for accurate diagnosis and appropriate management. Supplementary examinations yielded valuable data and reveal new signs. The patient needs life-long rehabilitation.

## Acknowledgments

The author thanks all those who participated in the data collection.

## Author contributions

**Data curation:** Wei Shen, Min Dai.

**Investigation:** Wei Shen, Min Dai.

**Methodology:** Qing Zhang.

**Supervision:** Hongsong Li.

**Visualization:** Wei Shen, Yunshan Su.

**Writing – original draft:** Wei Shen, Min Dai.

**Writing – review & editing:** Wei Shen.
